# Immune-stimulating Effect of *Lactobacillus plantarum* Ln1 Isolated from the Traditional Korean Fermented Food, Kimchi

**DOI:** 10.4014/jmb.2001.01038

**Published:** 2020-03-20

**Authors:** Hye Ji Jang, Hyung-Seok Yu, Na-Kyoung Lee, Hyun-Dong Paik

**Affiliations:** Department of Food Science and Biotechnology of Animal Resources, Konkuk University, Seoul 05029, Republic of Korea

**Keywords:** *Lactobacillus plantarum*, probiotics, heat-killed cells, immune-stimulating

## Abstract

This study aimed to determine the immune-stimulating effects of heat-killed *Lactobacillus plantarum* Ln1 (HK-Ln1) through the production of nitric oxide (NO) and pro-inflammatory cytokine achieved by inducing NF-κB and mitogen-activated protein kinase (MAPK)-signaling pathways in macrophages. HK-Ln1 showed higher NO and cytokine production compared to control (nonstimulated lipopolysaccharide); in addition, the expression of inducible nitric oxide synthase (iNOS) was induced through HK-Ln1treatment. The phosphorylation of IκB-α and p65 increased following treatment by HK-Ln1, which implicates IκB-α degradation and the translocation of p65 to nucleus. In addition, the phosphorylation of MAPKs, ERK 1/2, JNK, and p38 was induced following HK-Ln1 treatment.

Lactic acid bacteria (LAB) are widely known probiotics and para-probiotics used to improve gut condition, barrier function, and immunity [1-4]. LAB is reported to improve the immune system and decrease the risk of infection by bacteria, viruses, and other pathogens [5]. Although the mechanisms of immune system stimulation by LAB has not yet been fully understood, heat-killed probiotics as well as live probiotics are reported to have immune-stimulating effects [6]. Thus, the aim of this study was to evaluate the immune-stimulating effect of heat-killed *Lactobacillus plantarum* Ln1 (HK-Ln1) isolated from kimchi.

*L. plantarum* Ln1 isolated from kimchi and *L. rhamnosus* GG (LGG) were grown in lactobacilli MRS broth (BBL, BD Biosciences, USA) at 37°C for 15 h. LGG, used as the control strain, was obtained from Korean Collection for Type Cultures (Korea). To obtain heat-killed *Lactobacillus*, the cells were heated at 80°C for 30 min. The final concentration of heat-killed *Lactobacillus* was adjusted to 10^7^ and 10^8^ CFU/ml, respectively. To investigate the immune-stimulating effects of HK-Ln1, nitric oxide (NO) assay, semi-quantitative real time RCR, and Western blot assay were performed with some modifications [7, 8]. In addition, we used specific inhibitors such as MAPKs inhibitors (ERK 1/2, PD98059; JNK, SP600125; p38, SB203580) and NF-кB inhibitor (PDTC). NO is known to be involved in various physiological processes, such as nerve growth, neurotransmission, and regulation of cardiovascular pressure; furthermore, NO has been used for treatment of vascular disorders [9, 10]. It has been reported that NO plays an important role in immune response and host defense against invading pathogenic bacteria and viruses, as well as tumor cells [11]. NO produced by iNOS plays a physiological role in immune function after LPS stimulation to protect host cells [12]. This study demonstrated immune-stimulatory effect of HK-Ln1 compared to 10 ng/ml LPS. Generally, in order to evaluate the immune-stimulating other LAB, LPS concentration was performed at 1-10 ng/ml to minimize the cytotoxic effect [7, 12, 13]. NO production was examined in RAW 264.7 cells by NO assay. HK-Ln1 (10^8^ CFU/ml) showed the highest NO production (5.31 μM) compared to that of cell non-treated LPS (3.74 μM). NO production was higher in HK-Ln1 (10^8^ CFU/ml) at 5.30 μM than in LGG (Fig. 1A). NO assay is convenient tool for detection of immune response. Many studies have reported that probiotics can also stimulate the immune system, resulting in modulation of inflammatory mediators through cytokines that are responsible for the maintenance of the pathological process or immune response in a regulatory sense [14]. In a previous study, NO production of *Lactobacillus brevis* KCCM 12203P, *Lactobacillus paraplantarum* SC61, and *L. plantarum* 200655 was reported to be 21.83, 14.77, and 11.38 μM, respectively [7, 12, 13].

When external substances invade the body, macrophages modulate the immune system via production of cytokines such as interleukin (IL)-1β, IL-6, and tumor necrosis factor (TNF)-α. [12]. In a different study, it was reported that LAB promotes macrophages through the production of cytokines associated with immune response to foreign materials [12, 15]. HK-Ln1 showed immunostimulatory effects on the mRNA expression of iNOS, IL-1β, IL-6, and TNF-α (Figs. 1B-1E). In this study, to identify immune-stimulating activity of HK-Ln1, we researched by using in vitro anti-inflammatory mechanism through IL-6, IL-1β, and TNF-α, in macrophages or dendritic cells [16]. Many studies reported that Th2 cells produce IL-4, IL-6, IL-10, and TNF-α and regulate humoral immunity [17]. These have shown to stimulate inflammatory response to protect host cells. LPS has known as a direct inducing mediator for producing NO. However, HK-Ln1 is used as a substance that does not induce inflammation but induces immune responses in this study. Previous studies have shown that immunity is enhanced at low LPS levels when inducing inflammation [7, 12, 13]. In comparison with non-treated LPS, the cells cultured with HK-Ln1 (10^8^ CFU/ml) showed higher levels of the following cytokines: iNOS (3.44-fold), IL-1β (669.76-fold), IL-6 (10,697.39-fold), and TNF-α (5.93-fold). The mRNA expression of HK-Ln1 was higher than that of LGG at 10^7^ and 10^8^ CFU/ml. *L. plantarum* 200655 and *L. paraplantarum* SC61 increased the production of IL-1β, IL-6, and TNF-α [7, 12]. In addition, LAB isolated from kimchi increased the levels pro-inflammatory cytokines including IL-1β, IL-6, and TNF-α [18]. Next, we evaluated HK-Ln1 related to iNOS expression (Fig. 2A). Similar to LPS-treatment groups, the protein expression of iNOS was noticeably induced by HK-Ln1 treatment. This result was correlated with up-regulation of NO production and iNOS mRNA expression following treatment by HK-Ln1. The up-regulated pro-inflammatory signature of macrophages led to the hypothesis that HK-Ln1-mediated immune-stimulating effect might be associated with the regulation of NF-κB, a pro-inflammatory transcriptional factor. The phosphorylation of IκB-α and p65 was increased following treatment with HK-Ln1 which implicates IκB-α degradation and the translocation of p65 to the nucleus (Fig. 2B). Additionally, we verified the NF-κB activation mediated by HK-Ln1 through phosphorylation of IκB-α; p65 was slightly decreased when treated with PDTC, the NF-κB-specific inhibitor. NF-κB is known to be a predominant transcriptional factor that modulates pro-inflammatory gene expression. The current results were supported by the previous study demonstrating the translocated NF-κB induces the expression of pro-inflammatory mediators, including iNOS, COX-2, and cytokines [19, 20].

The MAPKs mediated intracellular signaling pathways contribute to the regulation of cellular functions, such as gene expression, differentiation, mitosis, apoptosis, and cell survival. MAPKs are crucially involved in transcriptional regulation of pro-inflammatory responses [21, 22]. In particular, p38 MAPKs have been reported to play a vital role in the response to cellular processes [23]. The phosphorylation of MAPKs, ERK 1/2, JNK, and p38, was markedly induced by HK-Ln1 in a dose-dependent manner (Figs. 3A and 3B). The treatment of MAPKs specific inhibitor slightly reduced the HK-Ln1-induced phosphorylation of ERK 1/2, JNK, and p38; furthermore, HK-Ln1 exhibited higher stimulating effect compared to LGG at equal concentrations. Similarly, *L. reuteri* ATCC PTA 6475 displayed immune-modulating properties in macrophages through the regulation of MAPKs and NF-κB [24]. Therefore, these results demonstrated that the immune-stimulating potential of HK-Ln1 was associated with the activation of the NF-κB and the MAPKs signaling pathway.

## Figures and Tables

**Fig. 1:**
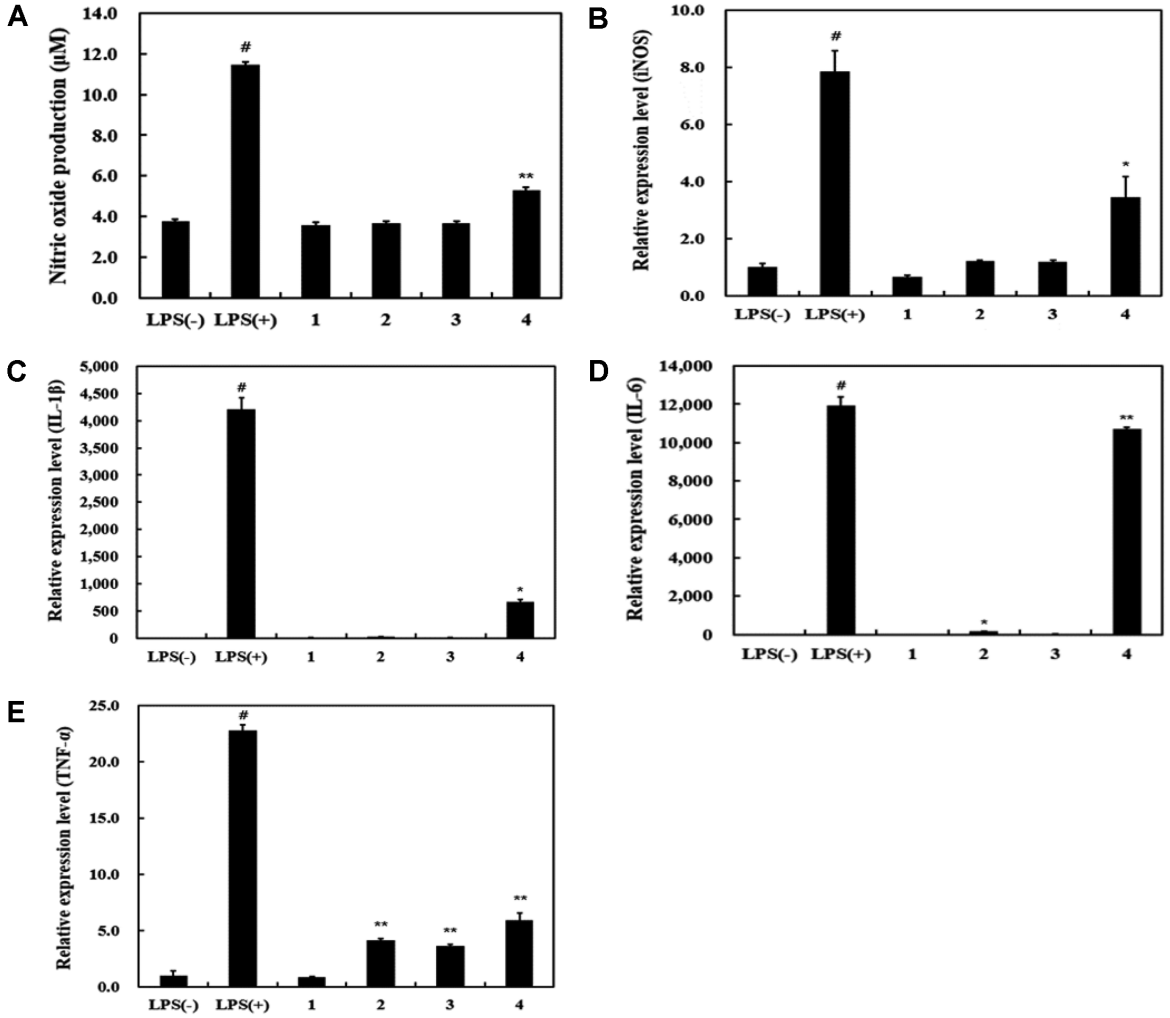
Effect of nitric oxide production and mRNA expression level of heat-killed *L. plantarum* Ln1 in RAW 264.7 cells. 1, *L. rhamnosus* GG (10^7^ CFU/ml); 2, *L. plantarum* Ln1 (10^7^ CFU/ml); 3, *L. rhamnosus* GG (10^8^ CFU/ml); 4, *L. plantarum* Ln1 (10^8^ CFU/ml); LPS (-), non-treated LPS; LPS (+), 10 ng/ml of treated LPS. (**A**) Nitric oxide; (**B**) iNOS; (**C**) IL-1β; (**D**) IL-6; (**E**) TNF-α. All values are represented as the mean ± standard deviation of triplicate experiments. #*p* < 0.01, respected to all samples; *p < 0.05, **p < 0.01, compared to non-treated LPS.

**Fig. 2:**
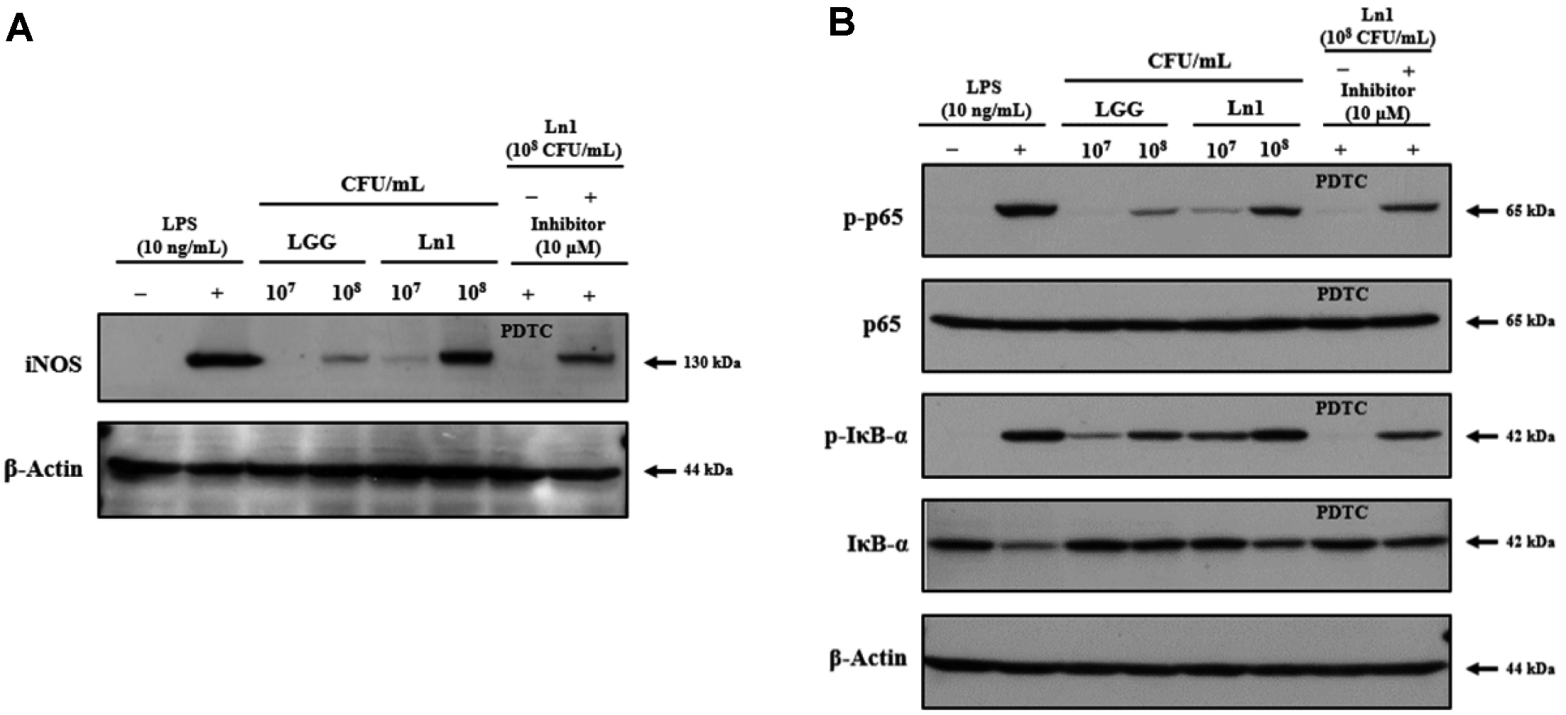
The effects of heat-killed *L. plantarum* Ln1 for iNOS expression and for NF-ĸB activation in RAW 264.7 cells. LGG, *L. rhamnosus* GG (10^7^ and 10^8^ CFU/well); Ln1, *L. plantarum* Ln1 (10^7^ and 10^8^ CFU/well). All groups were not treated with LPS. LPS (+) was treated with 10 ng/ml. NF-κB inhibitor was used PDTC with 10 μM. (**A**) Representative immunoblots of protein expression levels of iNOS and β-actin. (**B**) Representative immunoblots of protein expression levels of p-p65, p65, p-IκB-α, IκB-α, and β-actin. Results are representative of three independent experiments.

**Fig. 3:**
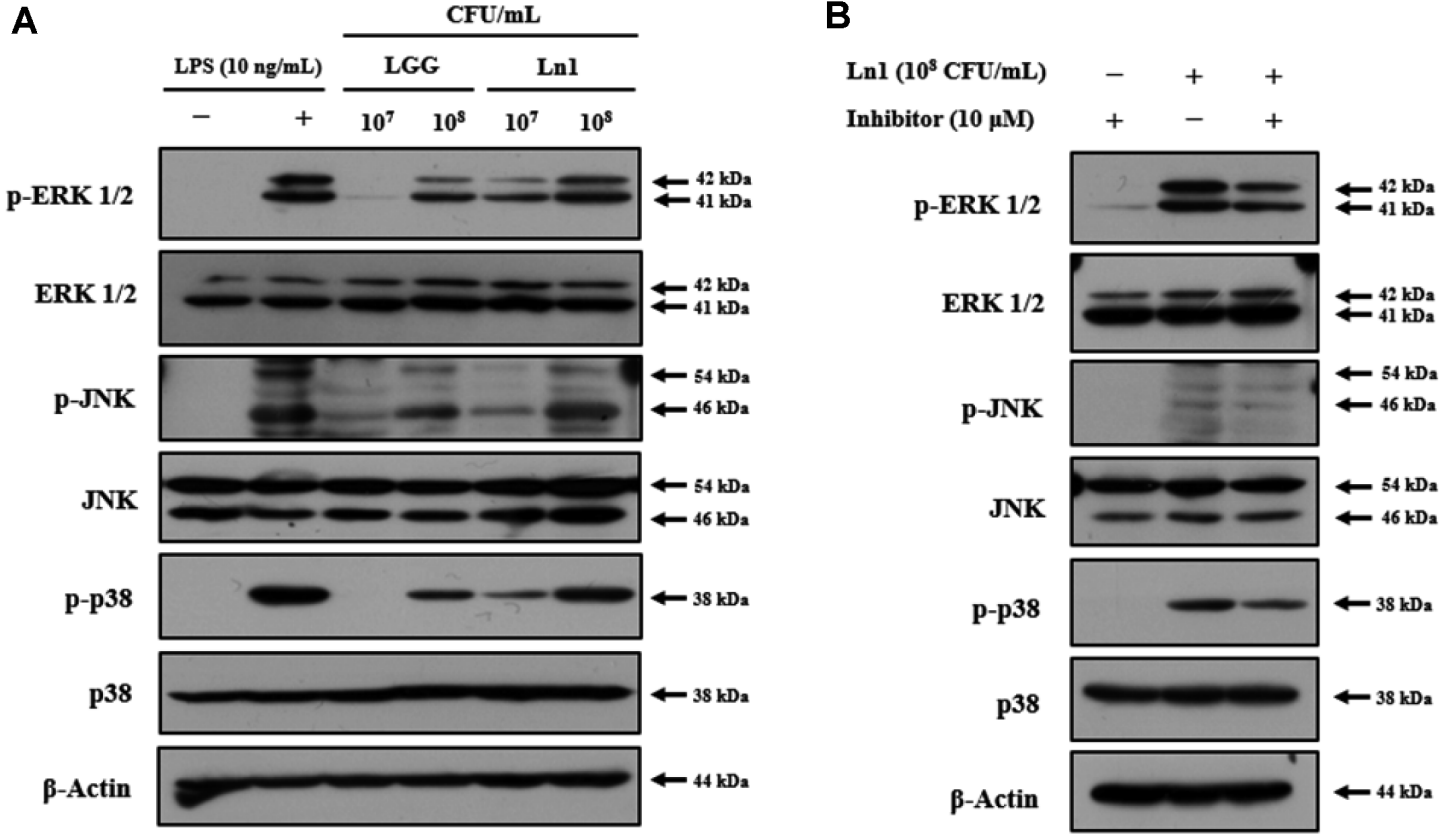
The effects of heat-killed *L. plantarum* Ln1 on phosphorylation of MAPKs in RAW 264.7 cells. LGG, *L. rhamnosus* GG (10^7^ and 10^8^ CFU/well); Ln1, *L. plantarum* Ln1 (10^7^ and 10^8^ CFU/well). All groups were not treated with LPS. LPS (+) was treated with 10 ng/ml. MAPK inhibitor was used PD98059 (p-ERK 1/2 and ERK 1/2) SP600125 (p-JNK and JNK), and SB203580 (p-p38 and p38) with 10 μM. (**A**) Representative immunoblots of protein expression levels of p-ERK1/2, ERK1/ 2, p-JNK, JNK, p-p38, p-38, and β-actin. (**B**) Representative immunoblots of protein expression levels of p-ERK1/2, ERK1/2, p-JNK, JNK, p-p38, p-38, and β-actin. Results are representative of three independent experiments.
